# Cancer Stem Cell Marker Endoglin (CD105) Induces Epithelial Mesenchymal Transition (EMT) but Not Metastasis in Clear Cell Renal Cell Carcinoma

**DOI:** 10.1155/2019/9060152

**Published:** 2019-03-19

**Authors:** Junhui Hu, Wei Guan, Libin Yan, Zhangqun Ye, Lily Wu, Hua Xu

**Affiliations:** ^1^Department of Urology and Institute of Urology, Tongji Hospital, Tongji Medical College, Huazhong University of Science and Technology (HUST), Wuhan 430030, China; ^2^Department of Pediatric Surgery, Tongji Hospital, Tongji Medical College, Huazhong University of Science and Technology (HUST), Wuhan 430030, China; ^3^Department of Molecular and Medical Pharmacology, David Geffen School of Medicine, University of California, Los Angeles, Los Angeles CA 90095, USA; ^4^Department of Urology, David Geffen School of Medicine, University of California, Los Angeles, Los Angeles CA 90095, USA

## Abstract

Clear cell renal cell carcinoma (ccRCC) is the most common histological subtype of kidney cancer. We previously reported that CD105(+) subpopulation in human ccRCC tumors possesses tumor cell self-renewal and chemoresistance capability. In this study, we showed that CD105(+) ACHN tumor cells exhibit epithelial mesenchymal transition (EMT) phenotype with high expression of mesenchymal marker N-cadherin and low expression of epithelial marker E-cadherin. They are more motile and invasive compared to the unselected parental ACHN tumor cells. The knockdown of CD105 by RNA interference led to the downregulation of N-cadherin and the upregulation of E-cadherin and reduced motility and invasiveness of CD105(+) cells. Overexpression of stem cell factor MYC in CD105 knocked down cells increased mesenchymal markers and cell motility. However, the CD105(+) population of tumor cells does not exhibit an increase metastatic potential *in vivo*. Findings from this study support that CD105 plays a functional role in maintaining cancer stem cell and EMT phenotype, with MYC as a common mediator for both of these traits. Our work suggests that the ability to metastasize does not coincide with the cancer stem cell or EMT function of CD105.

## 1. Introduction

Kidney cancer or renal cell carcinoma (RCC) is one of the 10 most common cancers in both genders, and clear cell renal cell carcinoma (ccRCC) is the major form, taking up 70~80% of all cases [1]. Patients with localized disease have excellent outcome after nephrectomy. However, one third of newly diagnosed patients will develop metastatic disease, and their outcome is extremely poor, with a 5-year survival rate of less than 10% [2]. A better understanding of the biology of metastatic ccRCC could be very helpful to improve the outcome of this disease.

Current views suggest that tumor-initiating cells could contribute to cancer metastasis. First, the self-renewal capability of tumor-initiating cells could seed and repopulate the growth of distant metastatic lesions, as previously reported [3]. Second, tumor-initiating cells often shared common markers and phenotypic behavior with cells that underwent epithelial-mesenchymal transition (EMT), as reported in CD44^high^/CD24^low^ breast cancer stem cell [4] and glioblastoma stem cells [5]. EMT is an embryonic program that allows polarized epithelial cells to migrate to distant site by the loss of cell-cell contact and the acquisition of more migratory and invasive phenotype. Concurrent with these phenotypic changes are an assortment of molecular changes, including loss of epithelial markers such as E-cadherin, and gain of mesenchymal markers such as N-cadherin and vimentin. These EMT changes have commonly been observed in clinical tumor specimen, including ccRCC [6], and they have been equated to tumor aggression or being metastatic in many cancer studies. But the direct causal relationship between EMT and metastasis *in vivo* has not been demonstrated. In fact, two recent prominent studies suggested that EMT is not necessary for metastatic spread but may play a critical role in resistance to chemotherapy [7, 8].

We recently reported that CD105 or endoglin is a marker for tumor-initiating cells that functions to maintain self-renewal and chemoresistance in ccRCC [9]. But little is known if CD105 is involved in ccRCC metastasis. Given the uncertainty of the relationship of cancer stem cell to EMT and metastasis noted, we decide to use the functional activity of CD105 to investigate these important and yet unresolved issues. In this study, we further interrogated the role of CD105 in EMT and metastasis by short hairpin RNA- (shRNA-) mediated knockdown of this gene. Our findings show that similar to its tumor-initiating capability, CD105 is necessary to maintain EMT phenotype via MYC. However, CD105 does not appear to contribute to ccRCC metastasis.

## 2. Materials and Methods

### 2.1. Ethics Statement

All the protocols in this study were approved by the Ethics Committee of Tongji Hospital affiliated with Tongji Medical School, Huazhong University of Science and Technology (HUST). All the mice in our experiments were kept in a specific pathogen-free (SPF) animal center in Tongji Medical School, and this study was designed to abide by the principles stated in the Declaration of Helsinki.

### 2.2. Cells, Plasmids, and Antibodies

The CD105(+) ACHN kidney cancer cell subpopulation is isolated and maintained as previously reported [9]. The shRNA plasmids for CD105 knockdown were constructed from pSicoR (Addgene, #11579) with target sequences of shENG1: 5′-GAAAGAGCTTGTTGCGCAT-3′ and shENG2: 5′-AACAGTCCATTGTGACCTTCA-3′, as previously reported [9]. Data presented were from knockdown with shEGN1, which is consistent with the results from shENG2. The ectopic overexpression plasmids of CDA, MYC, and NANOG were constructed based on the basic lentiviral vector modified from pSicoR (Addgene, #11579). Also, the labeling of EGFP or RFP in the cells for transwell assay was made by transfection of lentivirus from EGFP- or RFP-expressing plasmid with pSicoR (Addgene, #11579) backbone. 293T cells were purchased from ATCC.

For antibodies, anti-human C-myc (ab32072), E-cadherin (ab1416), and N-Cadherin (ab19348) antibodies were bought from Abcam (MA, USA); anti-human *β*-actin (AC026) antibody was bought from ABclonal (MA, USA); anti-human CD105(7508-1) and vimentin (2707-1) antibody were bought from Epitomics (CA, USA); and anti-human MHC-I (1913-1) were bought from Genetex (CA, USA). For flow cytometry antibodies, anti-human CD105 (130-112-321) conjugated with PE antibody was bought from Miltenyi (CA, USA) and was used for regular verification of CD105 expression in CD105(+) cell within 50 passages.

### 2.3. Migration Assay

In the transwell assay, 1 ml of expansion medium [9] supplemented with 50 ng/ml epidermal growth factor (EGF) was added in the well in a 24-well plate as chemoattractant. Then, transwell chambers (Cat #3422, Corning Corp., USA) were assembled in the plate, 1.0 × 10^5^ cancer cells were seeded on the chamber with 8 *μ*m pores on the membrane, and DMEM (low glucose) medium was added without growth factors up to 400 *μ*l in the chamber. After 24 hours, a cotton swab was used to swipe all the cells on the upper surface of the membrane and pictures at 5 random areas under 10x object lens were taken under the fluorescence microscope. Cell numbers were counted via Image J and analyzed via GraphPad Prism ver6.0.

### 2.4. Modified 3D Transwell Assay

In the modified 3D transwell assay, both CD105(+) cells, parental cells and shRNA-mediated CD105 knockdown cells, were lentivirally labeled with EGFP. On day 1, when the 24-well plate with transwell chambers was turned upside down, 1.0 × 10^5^ 293T cells that were lentivirally labeled with RFP were seeded on the bottom of the transwell membrane with a pore size at 2 *μ*m. Then, cells were incubated in RPMI-1640/10% FBS overnight to allow them to attach. On day 2, the 24-well plate was inverted to make it upright and 1.0 × 10^5^ EGFP-labeled cancer cells were seeded in 100 *μ*l Matrigel/RPMI-1640 (1 : 4 dilution ratio) above the transwell membrane. 1 ml RPMI-1640 without FBS or any supplement outside the transwell chamber was added and the cells were incubated for 1 hour at 37°C with 5% CO_2_ until Matrigel was solidified. Then, 300 *μ*l RPMI-1640 supplemented with 10% FBS and 50 ng/ml EGF was added upon the solidified Matrigel to serve as the chemoattractant layer; thus, the cancer cells can invade upward through Matrigel. After 48 hours, the transwell chamber is sent for confocal microscopic z-stack scanning in a total depth of 200 *μ*m and 5 *μ*m stepwise at 10x from the bottom, where the red 293T cells lie, upward to the top, and the green cancer cells lie. Volocity V8 was utilized to reconstruct the 3D images. Since red 293T cells' movement was blocked by the transwell membrane with a pore size only at 2 *μ*m, the position where the red cells are can serve as the reference where green cells start moving at time 0. Therefore, the bigger of the gap between the red and green cells is, the more motile and invasive the green cancer cells are since they migrated farther within the specified period of time. Supplementary Figure S1 shows the organization of cells and Matrigel in different layers.

### 2.5. Tail Vein Injection Model

12 Balb/c nude mice (5 weeks, female) were purchased from Charles River Corp. in China and randomly grouped into two groups, 6 for each. 1 × 10^6^ cancer cells for each mouse were counted and resuspended with 100 *μ*l precooled PBS for tail vein injection. Four weeks after the injection, all mice were euthanized after anesthesia by intraperitoneal injection of phenobarbital (80 mg/kg) and the lungs were dissected, weighed, and analyzed with GraphPad Prism ver6.0.

### 2.6. Quantitative Real-Time PCR (qRT-PCR) and Western Blot

For qRT-PCR in CD105(+) cells before and after treatment of TGF-*β* type I receptor kinase inhibitor LY-364947, 1 × 10^6^ cells of each type were seeded 1 day before the treatment in a 6-well-plate. On day 1, 50 nM of LY-364947 was applied on the cells and we waited for 48 hours to proceed with RNA extraction as mentioned below.

Total RNA was extracted via conventional phenol-chloroform extraction and reverse transcribed with a reverse transcription kit (Takara, Japan). The resultant cDNA was then examined by real-time RT-PCR kit using SYBR Premix Ex Taq from Takara (Japan). All primers were listed in Supplementary Table S1. The general procedure for western immunoblot is as previously described [9].

### 2.7. Statistical Analysis

All experiments were performed in triplicate unless otherwise stated. Data are presented as mean ± standard deviation (SD). Significance was determined by paired Student's *t*-test when there were two groups or by a one-way ANOVA when there were three or more groups. Figures were organized by using GraphPad Prism ver6.0. A *p* value cutoff of 0.05 was used to establish significance.

## 3. Results and Discussion

### 3.1. CD105 Is Necessary for ccRCC Self-Renewal and EMT Phenotype

**Figure 1:**
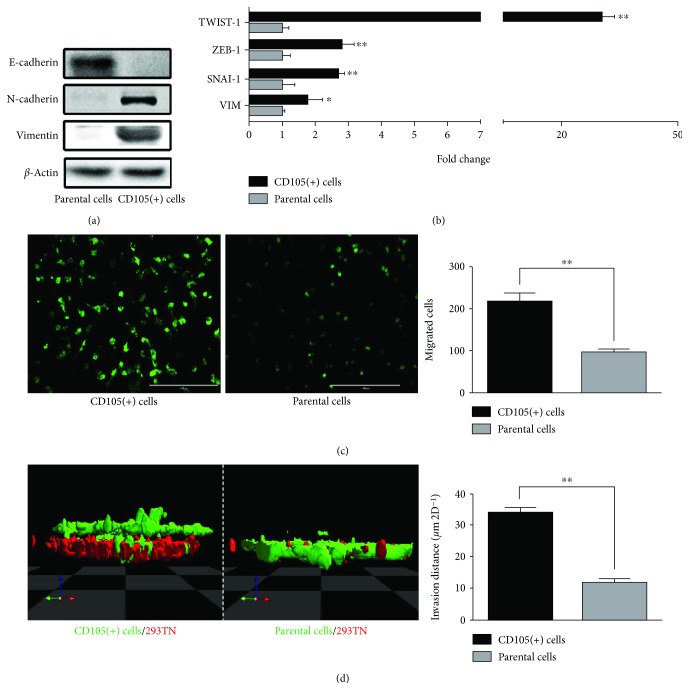
CD105(+) ccRCC tumor cells are mesenchymal cells with enhanced motility and invasion capability. (a) Western blot and (b) qRT-PCR of EMT markers of CD105(+) cells and parental cells. (c) Migration assay and (d) modified 3D transwell assay of CD105(+) cells and parental cells (^∗^ *p* < 0.05, ^∗∗^ *p* < 0.01).

The EMT has been associated with the acquisition of motility and self-renewal traits [10]. To gain a better understanding of the relationship between cancer stemness and metastatic potential, we first analyzed the EMT status of the CD105(+) tumor-initiating cells, isolated from ACHN renal tumors [9]. As shown in Figure 1, these CD105(+) cells are more mesenchymal than the parental cells, as they distinctly express elevated mesenchymal markers such as N-cadherin and vimentin and negligible epithelial marker E-cadherin, analyzed at the protein (Figure 1(a)) and RNA levels (Figure 1(b)). In further support of their EMT status, we analyzed the expression of EMT transcription factors (TFs) in the CD105(+) cells. The EMT TFs, such as TWIST-1, ZEB-1, and SNAI-1, are known to orchestrate EMT programs during development [11]. In particular, TWIST-1 is recognized to be associated with cancer metastasis [12, 13]. We observed over 20-fold higher expression of TWIST-1 in CD105(+) cells relative to parental cells (Figure 1(b)). ZEB-1 and SNAI-1 are also upregulated in CD105(+) subpopulation, albeit to a lesser extent than TWIST-1 (Figure 1(b)).

Next, we assess if the CD105(+) cells also acquired the functional EMT trait of increased cell motility in concert with their EMT genetic marker changes. A conventional 2D and a modified 3D transwell assay was used to evaluate cell migration and invasion through Matrigel, respectively. The motility of CD105(+) cells is significantly higher as the number of cells migrated through the transwell membrane with 8 *μ*m pores doubled that of the parental cells in the same period of time (Figure 1(c)). In the modified 3D transwell assay, a monolayer of tested cells was placed beneath a layer of Matrigel and their migration upward through Matrigel in response to a chemoattractant was assessed. The assay requires the cells to invade through the Matrigel barrier while counteracting the gravitational force. Corroborating the findings of the 2D assay, CD105(+) cells showed a greater than 2 times higher invasive capability in the 3D assay (Figure 1(d)).

**Figure 2:**
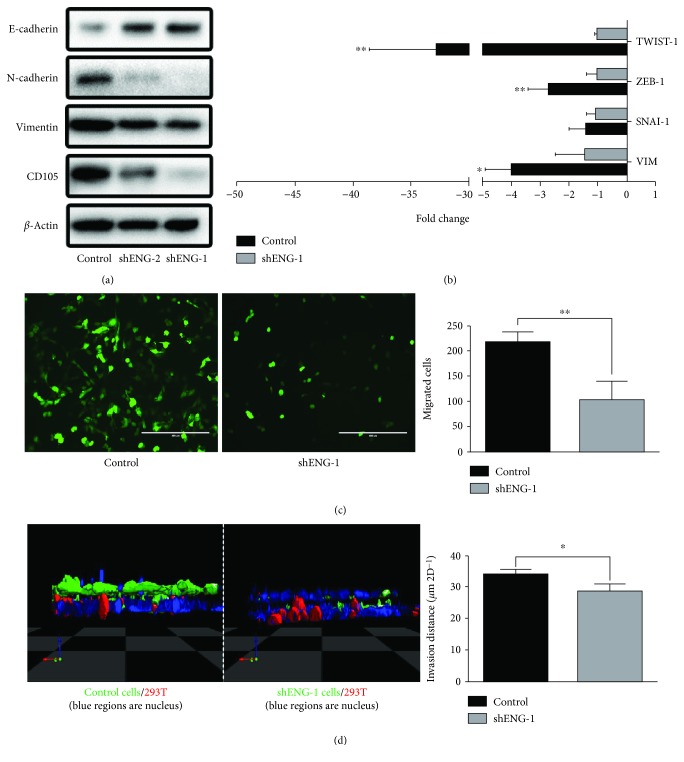
CD105 knockdown induces loss of mesenchymal markers and inhibition of motility and invasion. (a) Western blot and (b) qRT-PCR of EMT markers of CD105(+) cells and shRNA-mediated CD105 knockdown CD105(+) cells. (c) Migration assay and (d) modified 3D transwell assay of CD105(+) cells and shRNA-mediated CD105 knockdown CD105(+) cells (^∗^ *p* < 0.05, ^∗∗^ *p* < 0.01).

To further solidify the role of CD105 in EMT, we used two short hairpin RNAs (shRNA), denoted as shENG-1 and shENG-2, to knock down CD105 expression. As we previously reported, shENG-1 is more potent in downregulating CD105 than shENG-2 [9]. The loss of mesenchymal marker vimentin and N-cadherin and the increase of epithelial marker E-cadherin correlated closely with the level of CD105 downregulation (Figure 2(a)). Since shENG-1 is more effective in suppressing CD105 expression, all subsequent functional activity results presented will pertain to shENG-1. But similar results were obtained with shENG-2 (data not shown). The downregulation of CD105 expression by shENG-1 significantly lowered the expression of all 3 EMT TFs, especially TWIST-1 compared to control CD105(+) cells (Figure 2(b)). As expected, the motility and invasiveness of the CD105 knocked down cells were significantly depressed in our 2D migration assay (Figure 2(c)) and modified 3D assay (Figure 2(d)), respectively.

### 3.2. MYC but Not Nanog Can Reverse the Loss of EMT Traits Brought On by CD105 Downregulation

**Figure 3:**
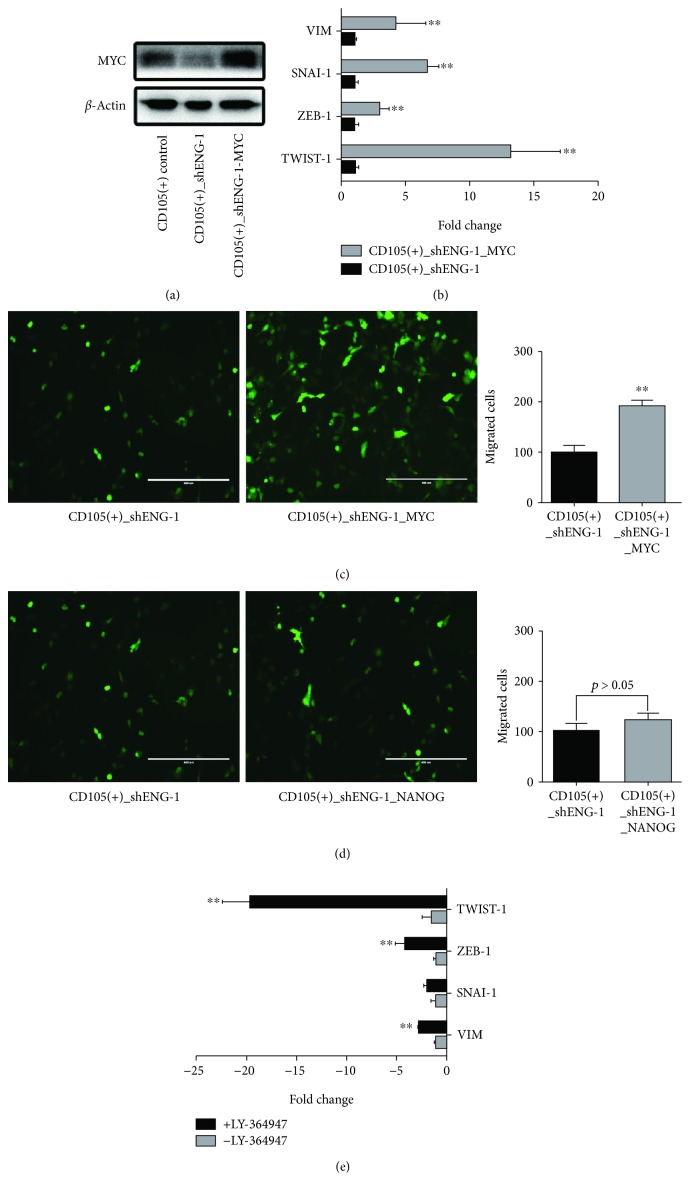
MYC overexpression can reverse this process. (a) Western blot of MYC in CD105(+) cells, shRNA-mediated CD105 knockdown CD105(+) cells, and MYC overexpressed cells. (b) qRT-PCR of EMT markers of shRNA-mediated CD105 knockdown CD105(+) cells before and after MYC overexpression. (c) Migration assay of shRNA-mediated CD105 knockdown CD105(+) cells and MYC overexpressed cells. (d) Migration assay of shRNA-mediated CD105 knockdown CD105(+) cells and NANOG overexpressed cells. (e) qRT-PCR analysis of EMT markers of CD105(+) cells before and after the treatment of TGF-*β* type I receptor kinase inhibitor LY-364947 (50 nM) (^∗∗^ *p* < 0.01).

As reported previously [9], the CD105(+) tumor-initiating cells expressed significantly elevated level of stemness factors such as Oct-4 Sox-2, KLF4, NANOG, and C-MYC. Furthermore, we demonstrated that MYC and NANOG are directly involved in maintaining cancer stem cell self-renewal function of the CD105(+) population. Here, we examined the functional contribution of MYC and NANOG to EMT. Consistent with our previous report [9], knockdown of CD105 by shENG-1 reduced MYC protein expression (Figure 3(a)). The reintroduction of MYC to the knockdown cells, denoted as CD105(+)-shENG-1_MYC, resulted not only in upregulation of EMT markers (Figure 3(b)) but also in increased cell motility compared to the knockdown cells (CD105(+)-shENG_1, Figure 3(c)). Overexpression of MYC is able to restore the loss of EMT functional activity conferred by CD105. However, unlike MYC, the reintroduction of NANOG into CD105(+)-shENG cells could not restore the loss of EMT activities (Figure 3(d)). Also, in order to confirm the effect of TGF-*β* on the EMT changes, a TGF-*β* type I receptor kinase inhibitor LY-364947 was applied on the CD105(+) cells and was found to inhibit the EMT markers including TWIST-1 significantly (Figure 3(e)).

### 3.3. CD105 Has No Impact on Metastasis *In Vivo*

**Figure 4:**
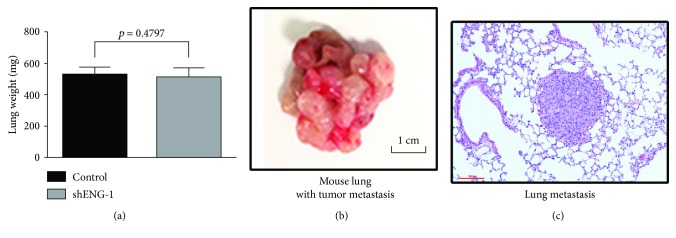
CD105 knockdown does not change the metastasis of ccRCC in the tail vein injection mouse model. (a) The lung weight analysis of mice received the CD105(+) cells and shRNA-mediated CD105 knockdown CD105(+) cells. Representative images of lung metastasis in both gross view (b) and H&E staining (c) established by tail vein injection of cancer cells.

With the clear *in vitro* EMT changes noted in the CD105(+) cells, we further examined their *in vivo* metastatic potential. The knockdown of CD105 in CD105(+)-shENG cells significantly decreased their growth potential as renal xenograft compared to CD105(+) cells in serial dilution tumorigenicity assay [9]. Previously [9], mice engrafted with CD105(+) or CD105(+)-shENG tumor revealed no metastatic lesion in distant organs such as the lung or liver in both of these groups, despite the presence of large primary tumors. Then, in this study, we assessed the ability of these 2 groups of tumor cells to establish lung metastases by direct intravenous injection of these cells. Both groups of mice, receiving either CD105(+) or CD105(+)-shENG cancer cells, develop extensive lung metastases with equal efficiency, as there is no statistical difference in their total lung weight (Figure 4(a)) or in the gross pathology (Figure 4(b)) or histology (Figure 4(c)).

Taken together, our results in this study demonstrated that in addition to governing a stemness phenotype, CD105 can also induce an EMT program with increased cell motility and invasion in the ccRCC tumor cells. The EMT and stemness traits controlled by CD105 share a common mediator in MYC, but they are distinct in that NANOG is known to be involved in self-renewal but not in the EMT program. However, our results showed that CD105 does not impact the metastatic potential of renal tumors *in vivo*.

CD105 is a homodimeric coreceptor of transforming growth factor *β* (TGF-*β*), an inflammatory cytokine [14]. TGF-*β* is often produced by immune cells such as tumor-associated macrophages (TAMs) [15], dendritic cells (DCs) [16], and regulatory T cells (Tregs) [14]. Extensive studies support that an inflammatory tumor microenvironment, such as that influenced by a high level of TGF-*β*, could induce EMT in tumor cells and promote tumor progression and metastasis [17]. However, very few reports have implicated the involvement of CD105 in EMT and metastasis [18, 19]. Our study here showed that CD105 is involved in the EMT and stem cell traits but not metastasis. What could account for this disconnect between the stemness, EMT phenotype, and the metastatic behavior? A possible explanation is that the proliferative potential of the CD105(+) cells is significantly reduced, with a higher portion of cells in G0 arrest, compared to the nonselected ACHN tumor cells [9]. Cells in EMT are also known to be growth arrested, as suggested by the “grow or go” hypothesis. This hypothesis postulates that in different phases of tumor progression, tumor cell motility and metastasis are dissociated spatially and temporally from active cellular growth [20]. It is energetically more economical for cancer cells to focus on either growth or motility sequentially and not simultaneously, as observed in many cancers [20–23]. Another concept suggests that cancer cells undergo EMT to acquire invasive capability need to reverse this process by mesenchymal-epithelial transition (MET) to achieve metastasis in distant organs [11, 24–26]. This requirement of plasticity of EMT stages to achieve metastasis is consistent with the dissociated growth and metastatic phase of cancer progression put forth in the “grow vs. go” concept. Our work here suggests that the growth-retarded state of EMT and cancer stem cell phenotypes observed in the CD105(+) cells could be interfering with their ability to establish metastasis *in vivo.*

Further investigation is also needed to dissect the role of TGF-*β*/CD105 and MYC in achieving EMT. A few studies have suggested that overexpression of MYC can induce EMT in a breast carcinoma model [26], as we have showed here for ccRCC. However, a greater number of studies support that TGF-*β* induces EMT and suppresses cell growth by direct binding of SMAD3 to the MYC promoter and downregulates MYC expression [27]. A possible explanation for this dichotomy might be due to the differential signaling mediated by the three different CD105 isoforms. The long form of CD105, or CD105-L which is the major form in the CD105(+) ccRCC cells [9], can promote cell growth via ALK1/Smad1/Id1 while the short form of CD105 or CD105-S functions oppositely [28, 29]. Detailed investigation into the mechanism of CD105-mediated regulation of cancer stemness, EMT, cellular proliferation, and metastasis is clearly warranted. Better understanding of these critical cancer phenotypes will help devise more effective treatment for the advanced and aggressive subtype of ccRCC.

## 4. Conclusions

CD105 shared a common mediator in MYC to induce cancer stemness and EMT traits in ccRCC cells. Although these two traits are often associated with aggressive and metastatic disease, CD105(+) cells do not exhibit increased metastatic potential.

## Data Availability

All the data used to support the findings of this study are included within the article and supplementary material files.

## Supplementary Materials

Supplementary Materials Supplementary Figure S1: schematic organization of cells and transwell inserts in the modified 3D transwell assay. Supplementary Table S1: primer sequences.
