# Supplementary material for: Cancer Stem Cell Marker Endoglin (CD105) Induces Epithelial Mesenchymal Transition (EMT) but Not Metastasis in Clear Cell Renal Cell Carcinoma
Source: Stem Cells Int. 2019 Mar 19;2019:9060152. doi: 10.1155/2019/9060152 (PMC6444238; doi:10.1155/2019/9060152)
Supplement: Supplementary Materials — Supplementary Figure S1: schematic organization of cells and transwell inserts in the modified 3D transwell assay. Supplementary Table S1: primer sequences. [file 9060152.f1.pdf]

Supplementary Figure S1

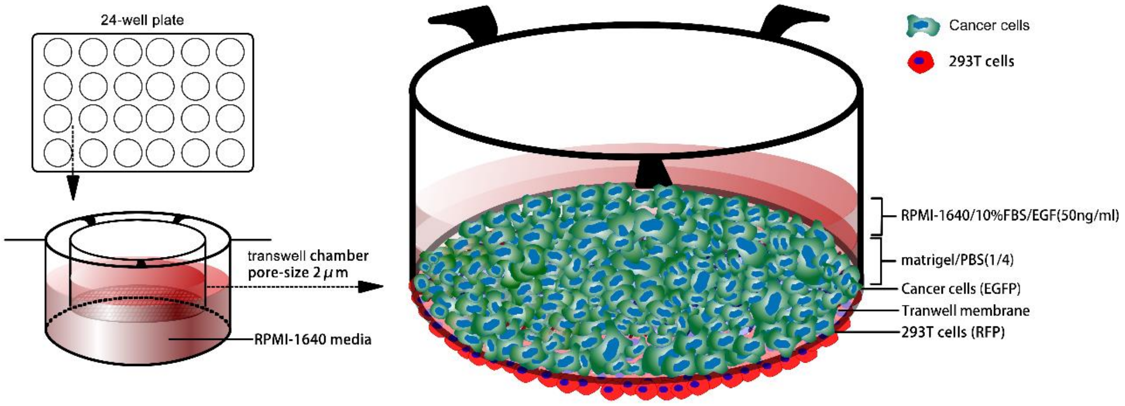

Figure Legend

Supplementary Figure S1. Schematic organization of cells and transwell inserts in the modified 3D transwell assay.

Supplementary table S1. Primer sequences

Table 1: Primer sequences

Primer Name	strand	sequence(5'-3')
TWIST-1	forward	GTCCGCAGTCTTACGAGGAG
	reverse	GCTTGAGGGTCTGAATCTTGCT
ZEB-1	forward	TTACACCTTTGCATACAGAACCC
	reverse	TTTACGATTACACCCAGACTGC
SNAI-1	forward	ACTGCAACAAGGAATACCTCAG
	reverse	GCACTGGTACTTCTTGACATCTG
VIM	forward	TGCCGTTGAAGCTGCTAACTA
	reverse	CCAGAGGGAGTGAATCCAGATTA
GAPDH	forward	CTGGGCTACACTGAGCACC
	reverse	AAGTGGTCGTTGAGGGCAATG
